# Supplementary material for: Trends in stroke admissions before, during and post-peak of the COVID-19 pandemic: A one-year experience from the Qatar stroke database
Source: PLoS One. 2022 Mar 24;17(3):e0255185. doi: 10.1371/journal.pone.0255185 (PMC8947388; doi:10.1371/journal.pone.0255185)
Supplement: S1 Table — (DOCX) [file pone.0255185.s001.docx]

**Supplementary Table 1: Baseline Characteristics of study population**

| Characteristic or Investigation | Total Patients  (n= 2639) | Arabs  (n= 1216, 46.1%) | South Asian  (n= 1423, 53.9%) | P- Value |
| --- | --- | --- | --- | --- |
| Age- Mean, years | 53.9 ±14.6 | 58.9 ±16.2 | 49.5 ±11.5 | < 0.001 |
| Sex - Males | 2005 (76.0) | 725 (59.6) | 1280 (90.0) | < 0.001 |
| Study Period |  |  |  |  |
| Pre-COVID Time (Sep 2019-Feb 2020) | 1157 (43.8) | 540 (44.4) | 617 (43.4) | 0.15 |
| Peak COVID Time (March-June 2020) | 531 (20.1) | 225 (18.5) | 306 (21.5) |  |
| Post-Peak COVID Time (July-Dec 2020) | 951 (36.0) | 451 (37.1) | 500 (35.1) |  |
| Final Diagnosis |  |  |  |  |
| Ischemic Stroke | 1218 (46.2) | 464 (38.2) | 754 (53.0) | < 0.001 |
| Transient Ischemic Attack | 254 (9.6) | 139 (11.4) | 115 (8.1) |  |
| Intracerebral Hemorrhage | 218 (8.3) | 53 (4.4) | 165 (11.6) |  |
| Cerebral Venous Sinus Thrombosis | 36 (1.4) | 9 (0.7) | 27 (1.9) |  |
| Stroke Mimic | 913 (34.6) | 551 (45.3) | 362 (25.4) |  |
| TOAST Classification (n=1232) |  |  |  |  |
| Small Vessel Disease | 506 (41.1) | 178 (38.1) | 328 (42.9) | 0.004 |
| Large Vessel Disease | 233 (18.9) | 80 (17.1) | 153 (20.0) |  |
| Cardio-Embolic | 321 (26.1) | 150 (32.1) | 171 (22.4) |  |
| Stroke of Determined Origin | 62 (5.0) | 18 (3.9) | 44 (5.8) |  |
| Stroke of Undetermined Origin | 110 (8.9) | 41 (8.8) | 69 (9.0) |  |
| NIHSS |  |  |  |  |
| NIHSS on admission (mean) | 4.1 ±6.3 | 3.5 ±6.1 | 4.6±6.3 | < 0.001 |
|  |  |  |  |  |
| Mild Stroke (NIHSS 4 or less) | 1959 (74.2) | 965 (79.4) | 994 (69.9) | < 0.001 |
| Moderate Stroke (NIHSS 5-10) | 352 (13.3) | 120 (9.9) | 232 (16.3) |  |
| Severe Stroke (NIHSS > 10) | 328 (12.4) | 131 (10.8) | 197 (13.8) |  |
| Intervention |  |  |  |  |
| Thrombolysis | 116 (4.4) | 43 (3.5) | 73 (5.1) | 0.05 |
| Door to Needle Time |  |  |  |  |
| Interventional Thrombectomy | 33 (1.3) | 12 (1.0) | 21 (1.5) | 0.26 |
| Outcome |  |  |  |  |
| Modified Rankin Score – At 90 days |  |  |  |  |
| 0 | 610 (58.4) | 307 (62.4) | 303 (54.8) | < 0.001 |
| 1 | 115 (11.0) | 32 (6.5) | 83 (15.0) |  |
| 2 | 100 (9.6) | 50 (10.2) | 50 (9.0) |  |
| 3 | 72 (6.9) | 31 (6.3) | 41 (7.4) |  |
| 4 | 72 (6.9) | 28 (5.7) | 44 (8.0) |  |
| 5 | 53 (5.1) | 31 (6.3) | 22 (4.0) |  |
| 6 | 23 (2.2) | 13 (2.6) | 10 (1.8) |  |
|  |  |  |  |  |
| Systolic Blood Pressure | 155.8 ±53.1 | 149.0 ±69.1 | 161.5 ±33.5 | < 0.001 |
| Body Mass Index | 28.2 ±5.7 | 30.3 ±6.5 | 26.5 ±4.2 | < 0.001 |
| Length of Stay | 4.1 ±5.4 | 3.6 ±5.3 | 4.6 ±5.5 | < 0.001 |
