# Supplementary material for: Trends in stroke admissions before, during and post-peak of the COVID-19 pandemic: A one-year experience from the Qatar stroke database
Source: PLoS One. 2022 Mar 24;17(3):e0255185. doi: 10.1371/journal.pone.0255185 (PMC8947388; doi:10.1371/journal.pone.0255185)
Supplement: Attachment — Submitted filename: PLOS one 2021 Response to reviewers.docx [file pone.0255185.s002.docx]

Response to Reviewers

Some minor points to consider:
1. was there an influence of vaccinations on the rise of cases?

**This is a good question but unfortunately we were unable to evaluate the effect of vaccinations on the rates of stroke in our study.**

2. it is stated that 98% of all cases are admitted. This seems to be the number for all hospitalized cases? Please clarify.

The Hamad General Hospital (HGH is a tertiary care facility in the State of Qatar. The Stroke Program with the thrombolysis and thrombectomy facilities for the Stroke of Qatar are located at the HGH. Almost all acute strokes (98%) in Qatar are transferred to the hospital for treatment. **We have made changes in the manuscript to make it clear for the readers.**

3. the overall young age and male preponderance needs special mentioning although the relative rates did not change over time. Also, why so few females seek hospital admission.

We have made changes in the manuscript to explain that more than 70% of the population in Qatar comprises of young age (less than 55 years), with 5:1 expatriate males/female ratio. The younger age and higher percentage of male patients reflects these demographics.

4. no mention of minorities or non-Qataris is a clear weakness of this study and should be addressed. Where more severe strokes as seen during the pandemic due to higher admission rates of non-Qataris?

We thank the reviewer for the comment. We have added a supplemental table to explain the “minorities and non-Qatari” demographics

5. the low mortality of around 1% needs explanation.

The low mortality is secondary to predominantly mild sub-cortical stroke in younger population. We have explained this in the results section

6. Table 1 should denote that p values are for group comparisons between before and after and not other groups in the table. Or was it p for trend?

We have made the correction in the legend of the table.

Reviewer #2: This is an interesting article examining trends in stroke admission 6 months before, 4 months during and 6 months after the COVID -19 pandemic.

One question - the authors state that the numbers of Afib patients was likely fewer due to fewer holter monitors being placed during the pandemic, however can you expand this thought? How many stroke patients are routinely diagnosed as having afib as an outpatient with a Holter Monitor? Presumably most patients with Afib would still be diagnosed from their telemetry monitoring.

We thank the reviewer for the question. We have made changes to the results section to explain that the lower atrial fibrillation rates are likely because of the younger age of the patients. We have previously reported this and have included the reference in the manuscript.
